# Intimate Partner Stalking/Pursuit: A Pathophysiology of Attachment Style

**DOI:** 10.1177/0306624X211010289

**Published:** 2021-04-24

**Authors:** Catherine J. Creamer, Christopher J. Hand

**Affiliations:** 1 Glasgow Caledonian University, UK

**Keywords:** attachment, stalking, vagal tone, intimate partner stalking, heart rate variability, intimate partner violence, obsessive relational intrusion, autonomic nervous system, anxiety, polyvagal theory

## Abstract

Approximately half of stalking victims were previously in an intimate relationship with the perpetrator, and attachment style is strongly correlated with intimate partner stalking (IPS). In the first study to investigate polyvagal theory in IPS, we examined 58 adult participants’ attachment style, sex, history of IPS, vagal tone activity (i.e., heart rate variability; HRV), and cognitive processing disruptions (i.e., Stroop performance) in either participants who wished a relationship or in those who wished to maintain a relationship post-break-up. Results showed that males were more likely to perpetrate IPS than females. Anxious-style participants were more likely to have perpetrated IPS, showed greater cognitive disruption and HRV than avoidant-style participants. Our results support theories that attachment is a biological imperative with neurobiological implications that can be indexed physiologically and cognitively. This study is the first to demonstrate a pathophysiology of attachment style to IPS, in a replicable way. IPS is discussed as reflective of disordered arousal and related to anxiety. Recommendations for further research and clinically-relevant interventions are presented.

## Introduction

In line with the difficulties in specific legislation, measuring the prevalence of stalking is problematic and varies from jurisdiction to jurisdiction. In the United States, the National Crime Victimization Survey (Baum et al., 2009) estimated that approximately 3.4 million Americans were stalked in the 12 months preceding the survey. According to the Crime Survey for England and Wales, 4.6% of women and 2.5% of men aged 16 to 74 were victims of stalking in 2019/2020. Difficulties tend to arise in the definition of the term “stalking.” Cupach and Spitzberg (2004) have defined another type of problematic behavior, which they term Obsessive Relational Intrusion (ORI). Broadly speaking, ORI is defined as a “pattern of repeated, unwanted pursuit and invasion of one’s sense of physical or symbolic privacy by another person, either stranger or acquaintance, who desires and/or presumes an intimate relationship” (p. 358). They suggest this differs from the term “stalking” in that the legal criteria for stalking in many jurisdictions includes repeated actions, victim fear and in some cases, intent to produce fear, with either strangers, or non-strangers (Dutton & Winstead, 2006, 2011). In ORI, the term “relational” is important in defining the phenomenon, since the main motivation is to initiate or maintain an intimate relationship, and not necessarily to produce fear (﻿Cupach & Spitzberg, 2004; Cupach et al., 2011). Nevertheless, there is significant variation in the literature regarding the definitions of stalking from both a legal and empirical perspective because of the highly subjective nature of the perception and emotional reaction as to what constitutes unwanted behavior (Patton et al., 2010). Notwithstanding the point on the subjective nature of fear and unwanted behavior, in the US, the Centers for Disease Control and Prevention and the Bureau of Justice Statistics have detailed definitions of stalking and recent research is rooted more so in definitions that include a pattern of behavior that instills fear in the victim. Given this divergence in the literature, for the purposes of this research, the term relational refers to intimate partner pursuit (IPS), either wanting to establish or to maintain an intimate relationship and the associated behaviors involved in this pursuit. Acknowledging the divergence in the literature relating to the term stalking, the terms IPS and pursuit/ORI are used interchangeably. However, it is this relational term that has driven the development of theoretical assumptions and thus drives the current research in testing these assumptions. This research is not so much concerned with the definition of stalking, but more the dimensions of pursuit behavior (McEwan et al., 2019) in those with disruptive infant attachment in later intimate relationships; desired or wished to maintain.

Whereas most stalkers are known to their victims, the number of people who report being pursued in the context of relationship pursuit represents approximately 46% of all stalking cases (Boehnlein et al., 2020; Metropolitan Police, 2020; Weller et al., 2013). This is likely to be an underestimation of the extent of the problem, given that individuals may be reluctant to disclose such experiences for a variety of different reasons. Further, despite no contact or restraining orders, prosecuting perpetrators of stalking does not always lead to the behavior stopping (Eke et al., 2011; McEwan et al., 2019). One significant difference between stranger stalking and intimate partner stalking (IPS) or intimate partner pursuit is the difference in the perpetrated rates of violence between these two groups, with intimate partner stalkers presenting with significantly higher rates of violence against individuals with whom they had previously shared an intimate relationship (Björklund et al., 2010; Mohandie et al., 2006). Although the relationship between stalking and violence is complex, stalking appears to precede the potential for lethality in intimate partner relationships. In as much as up to 85% of cases, victims were stalked prior to their murder by a former partner (McFarlane et al., 1999). In England and Wales, while male victims of homicide are more likely to be killed by a friend or acquaintance (39%), 51% of homicides against women are most likely to be committed by a partner or ex-partner, and the highest reported incidence of intimate partner violence (IPV) amongst women occurs in separated couples, indicating that women are at the greatest risk for lethal violence when ending a relationship.

Nevertheless, whilst stalking or pursuit can result in violence against a former intimate partner, it does not always result in violence—depending on the function of the stalking. IPS is dimensional rather than categorical, ranging from indirect pursuit, such as leaving letters, flowers etc., to more direct interpersonal behaviors and physical violence. As such, more research is required to determine the point at which IPS can evolve—for example, the transition from distal to proximal points (or vice versa) and the interaction, if any.

A more recent re-assessment of the link between stalking and IPV by McEwan et al., (2017) demonstrated that only 33% of stalking victims had been victims of IPV during their relationships. This finding is important for a variety of different reasons, none the least that theoretical models of IPV are insufficient in explaining stalking in intimate relationships and different risk factors may well be associated with different levels of stalking (McEwan et al., 2009; McEwan & Pathé, 2013; McEwan et al., 2011; Thompson et al., 2013).

Various motivations that drive stalking behaviors have been postulated in the literature (see Berenson et al., 2009; Cupach et al., 2011; Davis et al.,2012; Romero-Canyas et al., 2010), including rejection sensitivity (De Smet et al., 2012; Sinclair et al., 2011) which would suggest some ego or cognitive involvement.

Throughout the literature on intimate relationship and/or stalking, attachment theory, particularly the notion of stalking as a pathology of attachment (Meloy, 1998, 2007), appears very frequently (Davis et al., 2012; Dutton & Winstead, 2006, 2011; Hazan & Shaver, 1987, 1990, 1994; Storey et al., 2009; Thompson et al., 2013; Wigman et al., 2008; Youngs et al., 2013). There has been an increase in studies related to attachment, IPV and stalking behavior, with evidence to substantiate a difference in attachment styles between stalkers and non-stalkers (Patton et al., 2010; Tonin, 2004). In a study looking at bonding and adult attachment styles in different types of stalkers, MacKenzie et al. (2008) suggest that some of the research has provided support for the proposition that stalking may derive from a pathology of attachment.

Whilst attachment theory has generated a number of contrasting explanations of stalking, there is consensus that the development of an insecure attachment style impairs the individual’s ability to appropriately manage relationships in adulthood with a consequential propensity to stalk. To date, the mechanism for this apparent impairment in managing intimate relationships has not been established.

Given that intimate partner relationships closely resemble primary attachment figures in adulthood (Langhinrichsen-Rohling & Rohling, 2000; Langhinrichsen-Rohling et al., 2000), an understanding of the role of attachment style in relationships may be used to explain the motivations of perpetrators to pursue this attachment when the relationship has ended, or is one that they wish to establish.

Although there have been frequent associations between attachment style and stalking behaviors, this research has hitherto typically been correlational. Little has been stated or explored that discusses the relevant quantities (what is it about attachment theory that is relevant to pursuit behavior?) of attachment theory and how these quantities result in pursuit behavior. For the current study this begged the question, what does the term “pathology of attachment” mean? To understand this and thus find some way of measuring it, attachment theory was deconstructed to determine the relevant quantities that could be measured.

### Attachment Theory

Bowlby’s (1969, 1973) seminal work on attachment theory has been pivotal in providing psychological science with an understanding of the importance of a child’s early environmental experiences on their development, and has been influential in psychological research as a paradigm for explaining psychopathology. Hazan and Shaver (1987, 1990, 1994) developed a taxonomy of the attachment styles observed in adult relationships and showed how they affected the quality of those relationships. Four styles of attachment were identified in adults: secure, anxious-preoccupied, dismissive-avoidant, and fearful-avoidant (Hazan & Shaver, 1994).

Whilst a somewhat emerging area in contributing to the knowledge relating to criminal behavior, some studies from evolutionary psychology have looked at IPV and IPS (Duntley & Buss, 2012). In a review of the literature on stalking behavior and evolutionary psychology, Duntley and Buss (2012) hypothesized that a number of functions of stalking behavior have been shaped by evolutionary processes. For example, the authors suggest that stalking helps solve mating problems by acquiring a new mate or guarding an existing one. Whilst the authors do concede that their review is best viewed as a collection of hypotheses rather than empirical evidence, in terms of attachment theory, they suggest that future stalking research concentrate on the subset of stalking that represents a malfunctioning of evolved attachment adaptations, which evolutionary theory fully acknowledges. Interestingly, they have proposed that future research distinguishes between stalking that represents the “normal functioning” of evolved stalking adaptations, and stalking that represents a malfunctioning of other adaptations, such as those involved in attachment.

Bowlby’s (1969, 1973, 1979) attachment theory is based on evolutionary principles; Bowlby, like Darwin, was interested in “what animals do to maximize their chances for survival” (Bowlby, 1973, p. 8). If intimacy is an evolutionary requirement, what happens if this requirement is not met? No study has, to our knowledge, abstracted the relevant quantities of attachment theory that provide direction for further empirical research.

### Polyvagal Theory—Deconstructing the Link between Attachment Theory and IPS

In a Darwinian sense, the human nervous system is biased toward survival and social relationships are crucial for survival. Bowlby (1969) has demonstrated the profound psychobiological effects of disruptions in the mother-infant attachment bond. His seminal work described the quality of attachment to a primary caregiver as being an important variable in child development (and importantly, a guide to behavior in adult relationships). Bowlby’s work also provides the potential for examining how disruptions in the attachment process can result in emotional dysregulation and problematic behavior.

In recognizing the essential role of infant/primary caregiver relationship in the healthy development of the central nervous system and subsequent attachment style Porges (1995) has developed a model of mind-body interaction which is grounded in neurobiological research and accentuates the role of neural systems in emotional development and emotional responding. According to polyvagal theory (Porges, 1995, 2001, 2003, 2004, 2007, 2009; Porges & Furman, 2011) the neurotransmitter acetylcholine transmits feelings of “safety.” The central theme of polyvagal theory is that it outlines the mechanisms by which feelings of safety lead to social behavior. Whilst studying the evolution of the nervous system, Porges (1995) identified the vagus nerve as being central in changing the way in which science understands ANS functions. Porges (1995) posits that the autonomic nervous system (ANS) is composed of a hierarchical system of three sub-circuits. The phylogenetically newer neural circuit (social engagement system) can only be activated when the nervous system detects the environment as safe. When the environment is perceived as “unsafe” the more primitive systems are activated.

The ANS is to some extent outwith the conscious control of the individual, and non-optimal ANS activity can result in disruptions in cognitive processing tasks. This is further demonstrated by Vrticka and Vuilleumier (2012), who explain how individual attachment style can influence the way the individual encodes information and more importantly, the encoding of approach (safety) versus aversion (threat) tendencies in social interactions. In their review of neuroimaging data, Vrticka and Vuilleumier found in social interactions within intimate or close relationships, individuals with secure attachment style had stronger activation and activity in reward circuits, which contributed to approach-related attachment behavior. In those with insecure attachment, these individuals were more likely to show increased activity in brain regions that respond to general threats of a social kind, typically those associated with negative affect and fear responses, promoting defensive responses.

In the first and (so far) only study applying polyvagal theory to IPV, Umhau et al. (2002) looked at heart rate variability/heart rate variance (HRV) in perpetrators of IPV and control groups during a lying-and-standing exercise to examine the possibility that perpetrators have a disturbance in vagal control of heart rate during a posture challenge. Umhau et al. found that IPV perpetrators showed a different neural strategy in the regulation of heart rate, which they suggest may affect the perpetrators’ ability to modulate emotion and to control aggression. Whilst the study by Umhau et al. (2020) did not look at attachment style in IPV, the relevance of this to the current study is that IPS frequently occurs following relationships that have been characterized by IPV (Senkans et al., 2017)

Using polyvagal theory as a theoretical framework provides a clear rationale for the present study in investigating the neurobiological mechanisms of individuals with anxious attachment who engage in IPS. It is possible that pursuit behavior is a form of behavioral displacement; a substitute to calm the neural defence system. Parallels can be drawn with clinical disorders characterized by difficulties in expressing social behavior and social awareness, such as autism, anxiety disorders, obsessive-compulsive disorder (OCD), attachment disorders, and Post Traumatic Stress Disorder (PTSD); these disorders and their symptomology have been associated with invalid neuroception (Austin et al., 2007; Leckman et al., 1982).

### Aims and Objectives

The aim of the current study was to examine the role of participant attachment style on prior IPS perpetration, pursuit behaviors, and cognitive and physiological measures. We considered the co-variates of attachment style, participants sex, prior IPS perpetration history (yes/no), pursuit scale scores, Stroop Test performance, and HRV in response to processing different rejection scenarios (case vignettes).

Using polyvagal theory as a theoretical model, we tested the over-arching hypothesis that participants with anxious attachment would have lower vagal tone activity than participants with avoidant attachment in response to romantic break-up scenarios. This would indicate the activation of more primitive neural circuits (ANS) and fight/flight mechanisms. Given the previous research relating to the types of IPS and cognitive rejection sensitivity, we hypothesize that rejection sensitivity would be more prominent in individuals with anxious attachment (Sinclair et al., 2011). Given that non-optimal ANS activity can result in disruptions in cognitive processing tasks, this study also aimed to determine to what degree the type of rejection triggers an ANS response and therefore impairs cognitive processing. It is suggested in the literature that in individuals with anxious attachment, break-up situations depicting scenarios of ego related internal rejection “about you” will be depleted of self-regulation, as measured in this study by an ANS response and Stroop effect. We included more neutral types of rejection sensitivity such as a neutral condition “about nobody” to further test this.

In line with prior research, we anticipated that male participants would demonstrate more IPS/ORI and pursuit behaviors than female participants. We hypothesized that there would be an effect of participant attachment style on IPS/ORI perpetration, pursuit behaviors, and cognitive disruptions; anxious-style participants were predicted to demonstrate more ORI and pursuit behavior, and to experience greater cognitive disruption and atypical vagal variance compared to avoidant-style participants.

## Method

### Design and Participants

The current study employed a quasi-experimental design. Participants were randomly allocated to one of three Rejection Type case vignette conditions (*about me, about you, about no-one* [control]). We considered the co-variates of Attachment Style group (anxious, avoidant), personal history of ORI and typology of ORI, participant sex (female, male), and participant HRV and Stroop performance. We were able to establish Attachment Style and ORI groups on a post-hoc basis by administering the questionnaires following the experimental procedure.

Sixty-two (30 female, 32 male) participants from Glasgow Caledonian University (GCU) were recruited via e-mail on the GCU Students’ Association website and offered an incentive of a £5 cinema voucher. Given vagal variation between age groups, participants were included if they were between 18 and 35 years of age. Participants were excluded from the study if they were taking prescribed cardiac medication. Participants were instructed not to consume alcohol or other social drugs for 48 hours prior to the experiment. The study adopted zero-tolerance to incomplete datasets. As such, four participants were excluded following experimental procedures due to missing items on the IPS questionnaire (not indicating yes or no to ever having pursued) leaving a total of 58 participants (28 [48.3%] female; *M*_age_ = 22.11, *SD* = 2.97; 30 males [51.7%]; *M*_age_ = 21.87, *SD* = 2.27).

### Measures

#### Questionnaires

To minimize any potential impact on HRV, participants completed these questionnaires in situ following the experimental conditions.

#### Attachment style

Given previous research findings that it is not clear whether individuals behave in a similar way across all interpersonal relationships (Fraley et al., 2011) Attachment Style was measured as experiences in romantic relationships. Research suggests that more general questions about attachment lead to more socially desirable responses (Stein et al., 2002). Given that the study focus was to test the hypothesis that anxious attachment would lead to an increase in pursuit behavior, the Experiences in Close Relationships—Relationship Questionnaire (ECR-R; Fraley et al., 2011) was used to measure two types of attachment: attachment-related anxiety and attachment-related avoidance. The ECR-R questionnaire is a 36-item scale demonstrating very strong Cronbach’s α (>.90) and is suggested as the primary scale for measuring adult attachments in intimate relationships (Cassidy & Shaver, 2016). Participants rated each item a 7-point Likert-type scale where 1 = *strongly disagree* and 7 = *strongly agree*, and an example item was: “*When my partner is out of sight, I worry that he or she might become interested in someone else.*”. Participants were categorized as either anxious-type or avoidant-type based on their responses to this scale—participants’ relative scores on the Anxiety sub-scale and the Avoidance sub-scale were used to determine this classification (a participant who scored higher on Anxiety relative to their score on Avoidance would be classified as anxious-type, or vice versa).

#### Pursuit behavior

A modified version of the ORI-50 (Spitzberg, 2010) was used to measure pursuit behavior. The modified ORI-50 measured pursuit behavior in either stranger or acquaintance who desired and/or presumed an intimate relationship and former partners who have pursued a relationship once the relationship has broken down. Cupach et al. (2011), drawing together previous measures of unwanted relational pursuit (Cupach & Spitzberg, 2004; Langhinrichsen-Rohling et al., 2000), identified 31 scale items which assessed relational pursuit behavior. To minimize the risk of participant attrition in the current study, we selected from the 31 items the 17 which were identified by Cupach et al. (2011, p. 105) as “ordinary, milder relational pursuit behaviors.” The revised response scale comprised items Hyperintimacy (2 items), Mediated Contact (3 items), Interactional Contact (1 item), Surveillance (2 items), Invasion (2 items), Harassment and Intimidation (2 items), Coercion and Threat (3 items), and Aggression and Violence (2 items). Participants completed a non-equal-interval ordinal scale to indicate the frequency with which they had engaged in specific behaviors toward *any* previous/current partner (Never/Once/2–3 times/4–5 times/6–10 times/11–25 times/25 times, or more). Similar to the original and modified scales, our 17-item scale demonstrated good reliability (Cronbach’s α = .79). Participants were grouped into a nominal “ORI History” variable based on these responses—participants who answered “Never” to all items were identified as having no history of ORI behaviors (0), all other participants were coded as having an ORI history (1).

#### Case vignettes

To control for rejection sensitivity, case vignettes (see Appendix 1) showed key breakup situations with information held constant but manipulated within the vignette according to the type of breakup situation. These comprised depicting infidelity (about you), different life goals (about me) and a neutral situation (about nobody).

#### Vagal tone

HRV was measured via smart phone photoplethysmographic technology (Plews et al., 2017). Tonic HRV was measured as resting HRV. Phasic HRV was measured to show how the system reacts to stimulus-response between two different time frames. The smart phone photoplethysmographic technology does not provide beat-to-beat raw data but provides an overall HRV score through a calculation based on the time interval between each beat. Porges (2007) proposes that whilst HRV is not always valid in physiology research as a dependent variable, in psychophysiological research, production of respiratory sinus arrhythmia (RSA) is by involvement of neural regulation, so the focus is not on peripheral physiology. Therefore, under certain conditions RSA may be a marker of cardiac vagal tone and satisfy Grossman’s (2007) conditions.

#### Stroop test

A 40-item Stroop test (Stroop, 1935) was administered online via PsyToolkit (Stoet, 2017) to determine the impact of the experimental conditions on executive functioning and also to allow for a sufficient time frame in which to measure HRV. Following the recommendations of Laborde et al. (2017) on HRV experimental design, it was determined to limit the focus of reactivity to the research question and thus the case vignettes and not to use an Emotional Stroop Test; thus only congruent (e.g., black) and incongruent (e.g., blue) color words were used.

### Procedure

Participants were given a full instruction and information sheet prior to the experiment. Participants were randomly assigned to the experimental condition (case vignette). In keeping with the recommendations (Laborde et al., 2017) to allow for spontaneous breathing and natural vagal tone, there was no input into participants’ breathing such as telling participants to relax and so forth.

To reduce any impact on vagal tone other than that caused by the experimental conditions, participants were asked not to move throughout the measuring exercise. Each participant practiced the camera measurement. If their finger moved from the camera measurement the phone would “bleep” indicating the timer had stopped and would re-start once the finger was placed across the camera and flash. Participants sat on a comfortable chair with feet flat on the floor and legs uncrossed in front of the computer screen. They were informed that a short scenario would be read to them. They were then required to undertake a short task involving colors and words where they would have to identify the color of the word by clicking on the keyboard. Following this they would take part in an online questionnaire (HRV was not measured during this time) and then a short relaxation exercise.

**Figure 1.**
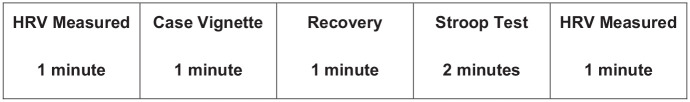
Experimental protocol.

Participants were asked to place their finger on the phone camera. Baseline HRV was recorded for 1 minute while resting and under standard conditions. The case vignette was read out slowly and 1 minute elapsed before the Stroop Test was administered on the computer screen. Following completion of the Stroop Test a one-minute period elapsed prior to recording of HRV for 1 minute. The experimental conditions were then declared over and the Stroop Test response times and HRV were recorded on the test sheet. A visual representation of the experimental protocol is outlined in Figure 1.

The online questionnaires were then administered on a separate screen window. The experimenter allowed participants to complete the questionnaires in privacy. Following the online questionnaires, participants were given the debriefing sheet and took part in a short progressive muscle exercise to reduce any emotional impact of the experimental conditions. Participants’ scores were recorded and then deleted from the phone application prior to testing of the next participant. The experiment received full approval from the Department of Psychology, Social Work and Allied Health Sciences’ Ethics Committee and conformed to British Psychological Society (2014) code of ethics.

### Statistical Analysis

Initial analyses of attachment style data enable us to create two groups of participants: 31 (53.45%) of participants were classified as anxious-type and 27 (46.55%) we classed as avoidant-type. The data from these 58 adult participants was included in the final analyses. In order to explore associations between participant sex, attachment style group, and personal ORI behaviors, a series of Pearson’s chi-square analyses were conducted. Further, in order to explore the independent effects of participant sex, attachment style group, and prior ORI history on Pursuit Scale data, a series of Mann-Whitney U tests were conducted. Additionally, we conducted two-way between-subjects analyses of variance (ANOVAs) on the independent and combined effects of rejection type case vignette, participant sex, attachment style group, and ORI history on Stroop test response times and HRV change (baseline vs. post-test).

## Results

### Chi-square Analyses

The first chi-square test considered the association between participant sex and prior ORI perpetration. It was expected that males would be more likely to have exhibited ORI behavior than females. Results are summarized in Table 1.

**Table 1.** Participant Sex and ORI Perpetration History.

			Male	Female
ORI history	No	Observed	12	19
		% within ORI group	39	61
		% within sex	40	68
	Yes	Observed	18	9
		% within ORI group	67	33
		% within sex	60	32

The association between participant sex and history of ORI behavior was significant (*χ*^2^ = 4.517, *df* = 1, *p* = .017; one-tailed); 60% of males sampled reported previous ORI behavior compared to only 32% of females. Of participants who reported a history of ORI behaviors, 67% were male whereas only 33% were female.

The second chi-square test considered the association between attachment style group and prior history of ORI. It was expected that with those with an anxious style would be more likely to have perpetrated ORI behaviors than those with an avoidant style. Results are summarized in Table 2.

**Table 2.** Attachment Style and ORI Perpetration History.

			Anxious	Avoidant
ORI history	No	Observed	12	19
		% within ORI group	39	61
		% within attachment style	39	70
	Yes	Observed	19	8
		% within ORI group	70	30
		% within attachment style	61	30

The association between attachment style and history of ORI behavior was significant (*χ*^2^ = 5.814, *df* = 1, *p* = .008; one-tailed). 61% of anxious-style participants reported a history of ORI behavior, whereas only 30% of avoidant participants reported such prior ORI behavior. Of participants who reported a history of ORI behaviors, 70% were anxious-style whereas only 30% were avoidant.

### Between-group Differences in Pursuit Behaviors

Anxious-type participants were expected to demonstrate higher pursuit scale scores/exhibit greater frequency of pursuit behaviors than avoidant-type participants. Table 3 shows the most-common (median) responses across groups. As the scales are essentially non-interval, the semantic label has been re-applied for illustration, reversing the numeric coding of responses which was necessary for inferential analysis.

**Table 3.** Median Pursuit Scale Response across Participant Sex, Attachment Style, and ORI History.

	Participant sex		Attachment style		ORI history	
	Male	Female	Anxious	Avoidant	No	Yes
Pursuit frequency	4–5 times	2–3 times	4–5 times	Once	Never	4–5 times

*Note*. Cells represent median values as labeled on the original scale.

When considering pursuit scores by participant sex, male responses indicated significantly more frequent pursuit behaviors than female responses (*U* = 302.000, *Z* = −2.010, *p* = .022; two-tailed). Pursuit behaviors across attachment style group were also examined, and it was observed that anxious-style respondents demonstrated more frequent pursuit behaviors than avoidant-style respondents (*U* = 230.000, *Z* = −3.216, *p* = .001; one-tailed). Unsurprisingly, those who had a prior history of ORI behavior scored higher on the Pursuit scale than those who had no such history (*U* = 496.000, *Z* = −7.140, *p* < .001; two-tailed)—this result is important in demonstrating additional validity of the pursuit scale data.

### Stroop Responses

A series of one- and two-way between-subjects ANOVAs were conducted to examine participants’ Stroop responses. Those with an anxious-type attachment style were expected to demonstrate longer Stroop responses than avoidant-type participants. For all analyses, Levene’s test of equality of error variances was satisfied (all Levene’s statistics <3.172, all significance values >.080). Descriptive statistics are shown in Table 4.

**Table 4.** Mean Stroop Response and Heart Rate Variability across Groups and Conditions.

	Participant sex		Attachment style		Rejection type case vignette (CV)			ORI history	
	Male	Female	Anxious	Avoidant	CV1	CV2	CV3	No	Yes
Stroop response	92.43 (14.76)	87.46 (16.08)	96.71 (17.18)	82.73 (8.38)	93.90 (17.03)	85.00 (13.99)	91.71 (14.47)	87.65 (13.05)	92.78 (17.72)
ΔHRV	7.11 (1.10)	7.28 (1.18)	6.85 (0.99)	8.26 (0.95)	7.02 (1.06)	7.19 (1.35)	7.15 (1.19)	8.11 (1.05)	6.79 (1.08)

*Note*. Mean figures rounded to 2DP; SDs in brackets. Stroop Response in seconds. ΔHRV = change in Heart Rate Variation between baseline and post-test, measured in percentage beats per minute.

Analyses revealed a non-significant effect of participant sex on Stroop response time (*F*[1,56] = 1.506, *MSE* = 237.399, *p* = .255). However, there was a significant effect of attachment style on mean Stroop response times; those with anxious-type attachment demonstrated longer response times (96.71) than those with avoidant-type attachment style (82.37) (*F*[1,56] = 15.552, *MSE* = 190.798, *p* < .001, η_p_^2^ = .217). The rejection type case vignettes had no bearing on Stroop response times (*F*[2,55] = 1.892, *MSE* = 232.242, *p* = .161). Participant ORI history had no effect on mean Stroop response times (*F*[1,56] = 1.604, *MSE* = 236.996, *p* = .211).

We additionally considered the possibility of interactive relationships between variables. We conducted a series of two-way ANOVAs to examine the relationships between attachment style and participant sex, attachment style and rejection type case vignette, and attachment style and ORI history. These analyses were performed to allow us to explore the independence of the effect of attachment style, and to ensure that this effect was not being influenced by an unseen underlying relationship with another variable. Across all two-way ANOVAs, consistent main effects as those yielded by the one-way ANOVAs were found, with no evidence of any interactions between these variables on Stroop response times (all *F*s < 1).

Finally, we considered the possibility of ORI history × participant sex and ORI history ×rejection type case vignettes on Stroop response data; all main effects were consistent with the one-way ANOVAs, and there was no evidence whatsoever of interactive relationships (both *F*s < 1).

### Heart Rate Variance (HRV)—Baseline versus Post-test

Individuals with anxious-style attachment will demonstrate lower vagal tone variance (as indexed by HRV) than those with an avoidant attachment style. An initial set of analyses examined participants’ baseline heart rate data and found no differences across groups (all *F*s < 1). A series of one- and two-way between-subjects ANOVAs were conducted to examine participants’ HRV (baseline vs. post-rejection vignettes). For all analyses, Levene’s test of equality of error variances was satisfied (all Levene’s statistics < 0.755, all significance values > .475). Descriptive statistics are presented in Table 4.

Analyses revealed a non-significant effect of participant sex on HRV (*F*[1,56] = 1.108, *MSE* = 1.293, *p* = .297). However, there was a significant difference between attachment style groups in terms of HRV; those with anxious-type attachment demonstrated a smaller HRV (Δ6.85%) than those with avoidant-type attachment style (Δ8.26%) (*F*[1,56] = 32.369, *MSE* = 0.836, *p* < .001, η_p_^2^ = .366). The rejection type case vignettes had no effect on HRV data (*F* < 1). Participant ORI history had a significant effect on HRV measurements; those with a history of ORI behavior demonstrated smaller HRV (Δ6.79%) than those who had no history of IPS (Δ8.11%) (*F*[1,56] =  8.984, *MSE* = 1.137, *p* = .004, η_p_^2^ = .138).

As with our Stroop analyses, we considered the possibility of interactive relationships between variables. We conducted a series of two-way ANOVAs to examine the relationships between attachment style and participant sex, attachment style, and rejection type case vignette, and attachment style and ORI history on HRV data. Across all two-way ANOVAs, we found consistent main effects as those yielded by the one-way ANOVAs, with no evidence of any interactions between these variables on HRV data (all *F*s < 1).

Finally, we considered the possibility of interactions between the effects of ORI history and participant sex, and ORI history and rejection type case vignettes on HRV. The two-way ORI history × participant sex interaction revealed consistent main effects to the one-way ANOVAs, but no significant interaction (*F*[1,54] = 2.995, *MSE* = 1.115, *p* = .089). The two-way ANOVA of ORI history × rejection type case vignette generated main effects that were consistent with the one-way ANOVAs, and there was no evidence whatsoever of an interactive relationship (*F* < 1).

## Discussion

Using polyvagal theory as a theoretical model, we tested the over-arching hypothesis that participants with anxious attachment would have lower vagal tone activity than participants with avoidant attachment in response to romantic break-up scenarios. The current study investigated adult participants’ attachment style, biological sex, history of ORI, vagal tone activity (i.e., heart rate variability; HRV), and cognitive processing disruptions (i.e., Stroop performance) in a controlled quasi-experiment. We anticipated that participants who identified as males be more likely to self-disclose IPS/ORI and demonstrate greater evidence of pursuit behaviors in relation to intimate partners; both of these hypotheses were upheld by our analyses. Additionally, we anticipated a clear relationship between participant attachment style and history of performing acts of IPS/ORI, pursuit behaviors, and cognitive and physiological responses. As hypothesized, we found that participants with anxious-style romantic attachments were more likely to have committed acts of IPS/ORI, and showed greater cognitive disruption (Stroop performance) and lower vagal tone variation (as indexed by HRV) following exposure to rejection case vignettes (see Appendix 1).

### Attachment Style, Pursuit Behavior, and Psycho-physiological Response

The purpose of our study was to test Polyvagal Theory as applied to romantic Attachment Style and pursuit behavior in response to scenarios depicting the ending of a relationship. The results of the study demonstrated a pattern of significant and medium-to-large effects, indicating that attachment style is impactful in relation to IPS/ORI and can be explained via reference to polyvagal theory and lower vagal activity.

Consistent with the extant literature on IPS and ORI, individuals with anxious attachment were found to be more likely to disclose IPS/ORI behavior than those with avoidant attachment. Individuals with anxious attachment show lower vagal activity (measured by HRV) when exposed to situations depicting relationship break-up, regardless of the type of break-up scenario, suggestive of the activation of the ANS. Previous studies (Sinclair et al., 2011) suggested that rejection sensitivity would be more prominent in individuals with anxious attachment, who would be depleted of self-regulation when exposed to break-up situations depicting scenarios of internal rejection “about you,” indicating some ego involvement. The present study marks a crossroads in this domain, demonstrating that pursuit behavior may have biological underpinnings. Similar to the rejection sensitivity and the rejection-hostility link (Romero-Canyas et al., 2010) the results suggest rejection sensitivity may function as a defensive motivational system.

The hypothesis that individuals who engage in (or have previously engaged in) IPS / ORI would have significantly different HRV is supported. The finding of differences in HRV those individuals who reported IPS/ORI is of interest because mediated contact is defined as technological or electronic contact, such as phone and e-mail and both males and females reported the same levels of mediated contact. These results indicate that those individuals with anxious attachment who engaged in mediated contact had less of a “vagal brake” when presented with stimulus depicting rejection indicating that activation of an ANS response.

This is further supported by analysis of the Stroop response times in perpetrators of IPS/ORI which demonstrated that this group took significantly longer in the Stroop test, supporting the hypothesis that the activation of the ANS impeded executive functioning. Although it is not possible to perform robust statistical analysis on subsets of the data based on IPS/ORI sub-scales (due to small cell sizes), some interesting patterns did appear (albeit superficially). For example, whilst only two participants reported aggression and violence as part of their history of IPS, these individuals took significantly longer on the Stroop test. It may be that more aggressive/intrusive ORI, such as surveillance and aggression and violence, correspond to reduced cognitive functioning—however, this must be explored in more detail in future studies.

There was no evidence of lower *baseline* vagal tone activity in individuals with anxious attachment. Whilst polyvagal theory postulates that low levels of RSA modulation indicate difficulties in social and emotional regulation and some clinical disorders (Austin et al., 2007; Eisenberg et al., 2000; Leary & Katz, 2004; Movius & Allen, 2005), it is assumed that this would result in lower baseline vagal activity between the different attachment styles and between perpetrators and non-perpetrators of IPS/ORI, which was not the case in the present study. This could be due to a number of factors, the most important one being that there will be individual differences in vagal activity due to cardiovascular health which was not controlled for in the study. However, the important result is not so much baseline vagal activity but the significant change in vagal activity across attachment style groups as a result of the introduction of rejection stimulus.

The findings of the present study support Polyvagal Theory as a method of determining whether there is a pathophysiology for IPS/ORI that could be further researched. The results suggest that an activated ANS is cognitively impenetrable (Porges, 2015). In terms of attachment theory, Bowlby (1969) discussed an infant’s attachment as a precondition for *survival*, not *happiness*. The literature on IPS/ORI to date has not accounted for this critical component when extracting theoretical assumptions from the correlation between attachment theory and IPS behaviors. The results of the current study demonstrate that in scenarios depicting abandonment, a defensive physiological state arises when the myelinated vagus is reduced and in some cases withdrawn.

### Limitations and Future Directions

Cyberstalking behavior was not measured in the version of the ORI scale used. However technology has advanced since the original version of the ORI and it is possible that when participants indicated surveillance type behaviors on the ORI they may well have been indicating cyberstalking. In light of the finding that mediated contact and surveillance type behaviors appear to increase parasympathetic activity, for future research, it is important to take into account the research on “cyberstalking” and therefore allow for the hypothesis that for participants with anxious attachment, cyber-pursuit could be their method of choice for IPS. It is a tenable proposal that mediated contact and surveillance function as a form of reassurance.

In addition to the limitations discussed herein, the design of the study may have had some limitations in terms of the rejection stimuli being verbal and not visual. There is some evidence in the literature that visual stimuli are more effective in moderating the effects of HRV and subsequent affective processing (Palomba et al., 2000). Replication of the present study using visual stimuli, such as video clips to investigate the impact of rejection types would serve to further test the hypothesis that internal rejection types “*about you*” are more likely to result in a decrease in vagal tone.

The use of Rejection Stroop (Sebastian & Ahmed, 2018) as a dependent variable would also greatly enhance the methodological validity of future studies. For future research in this area it is important to avoid subjective tests which detract from an understanding of the underlying mechanisms that drive IPS behavior.

We deliberately recruited a balanced sample of male and female participants, to be representative of the general adult population. However, we did not consider psycho-social dimensions of gender-sex; future research may wish to incorporate subjective measures of participants’ gender-sex identity (masculinity, femininity, androgyny; e.g., Bem, 1974). Furthermore, our participants identified as heterosexual—this of course limits the results of our study to that community; future research could either look at a single gender-sex sample who report same-sex attraction, or indeed, with a large enough sample, between-group comparisons could be made across gender-sex groups and sexual identity/orientation communities (i.e., LGBTQ+).

The scale we used and adapted asked participants if they had ever pursued a relationship; either trying to initiate one or once it had ended, and then identify the level of pursuit. We did not differentiate between trying to initiate a relationship or pursue one that had ended, which limits the results somewhat. Future studies could focus the research question on either one of these as there may be further differences in vagal tone activity between these two groups. It would be important in this context to either find, or develop a scale that reliably measures this (see McEwan et al., 2019). It would also be important to understand the legal definitions of stalking when developing scales of this nature and not to label behaviors as ordinary or minor, but to take reference from the emerging empirical literature on risk assessment in IPS. There is no evidence to date to suggest that a random seemingly benign behavior, such as leaving flowers, could not escalate into more serious behaviors such as threats and physical/sexual violence.

### Conclusion

The overall aim of this study was to test polyvagal theory as applied to Attachment Style and IPS/ORI and to elucidate the relationship between HRV and these variables in response to scenarios depicting the ending of a relationship. The hypothesis that perpetrators of IPS/ORI and those with anxious attachment will have lower vagal tone activity than participants who reported no IPS/ORI in response to internal rejection stimulus was supported, although rejection stimulus type did not appear to impact on the level of HRV. Whilst there are limitations to the study, the overall conclusions of the study support the hypotheses. The difference in HRV between romantic attachment styles indicated the differential activation of more primitive neural circuits (ANS) and fight/flight mechanisms in those with anxious attachment. The study has demonstrated that attachment style has neurobiological implications as measured by vagal tone that has the potential to explain IPS behaviors as anxiety-related and functional to reduce anxiety.

Despite methodological implications and limitations, this study provides support for the tenable proposal that attachment fears and anxiety may be key biological, cognitive, and emotional contributors to the behaviors seen in ORI/IPS. Future research could add value to this by looking at HRV in those already convicted of ORI/IPS, which could contribute to the growing literature on ORI/IPS and the link between attachment style. Whilst the aim of this study was to deconstruct attachment theory and thus establish a pathophysiology of disrupted attachment in ORI/IPS, the potential level of violence in IPS with disrupted attachment has not been established, but would be useful research question in future studies using HRV as a variable. As Bowlby (1969, 1973) suggested, fear and anger arise from the same place. This study has provided a novel methodological platform grounded in theory to undertake further research that also provides for the application and testing of more clinically-relevant interventions.

## Appendix 1

### Case Vignettes

#### Vignette one (*About You*)

Martin and Molly have been a couple for 2 years and moved in together 1 year ago. Their relationship has been happy, and Martin considers that this is “the real thing.” Recently Molly has become quiet and appears distant. When Martin asks her about this she tells him that work is going through a stressful period. Molly has also started to come home late stating that she has a number of work deadlines. One Saturday morning, Molly asks Martin to sit down as she has something to discuss with him. Molly looks agitated. Molly explains that she has met somebody else at work. She says that their relationship had been platonic but a few months ago this had changed. Molly says she is so sorry and she never wanted to hurt Martin. Martin asks if she loves him and Molly says she does.

#### Vignette two (*About Me*)

Martin and Molly have been a couple for 2 years and moved in together 1 year ago. Their relationship has been happy and Martin considers that this is “the real thing.” Recently Molly has become quiet and appears distant. When Martin asks her about this she tells him that work is going through a stressful period. Molly has also started to come home late stating that she has a number of work deadlines. One Saturday morning, Molly asks Martin to sit down as she has something to discuss with him. Molly explains that she has been thinking a lot recently about her life and her goals and that she has handed in her notice at work. She tells Martin that she would like to take a break in their relationship and to travel before it is too late.

#### Vignette three (*About Nobody*)

Martin and Molly have been going out together for 4 months. Martin really feels like this could be “the real thing” and when out to dinner one evening, tells Molly that he is serious about her. Molly tells Martin that she does not feel the same, that the chemistry is not there for her and she can’t manufacture an emotion. She thinks the best thing to do is to break up.
